# Supplementary material for: Evidence of strong and mode-selective electron–phonon coupling in the topological superconductor candidate 2M-WS2
Source: Nat Commun. 2024 Jul 24;15:6235. doi: 10.1038/s41467-024-50590-9 (PMC11266404; doi:10.1038/s41467-024-50590-9)
Supplement: Supplementary file 1 — Supplementary Information [file 41467_2024_50590_MOESM1_ESM.pdf]

Supplementary Material of

Evidence of strong and mode-selective electron–phonon coupling in the topological superconductor candidate 2M-WS_2

This file includes

Supplementary Figures 1-13

Supplementary Tables 1-3

Supplementary Note 1-2

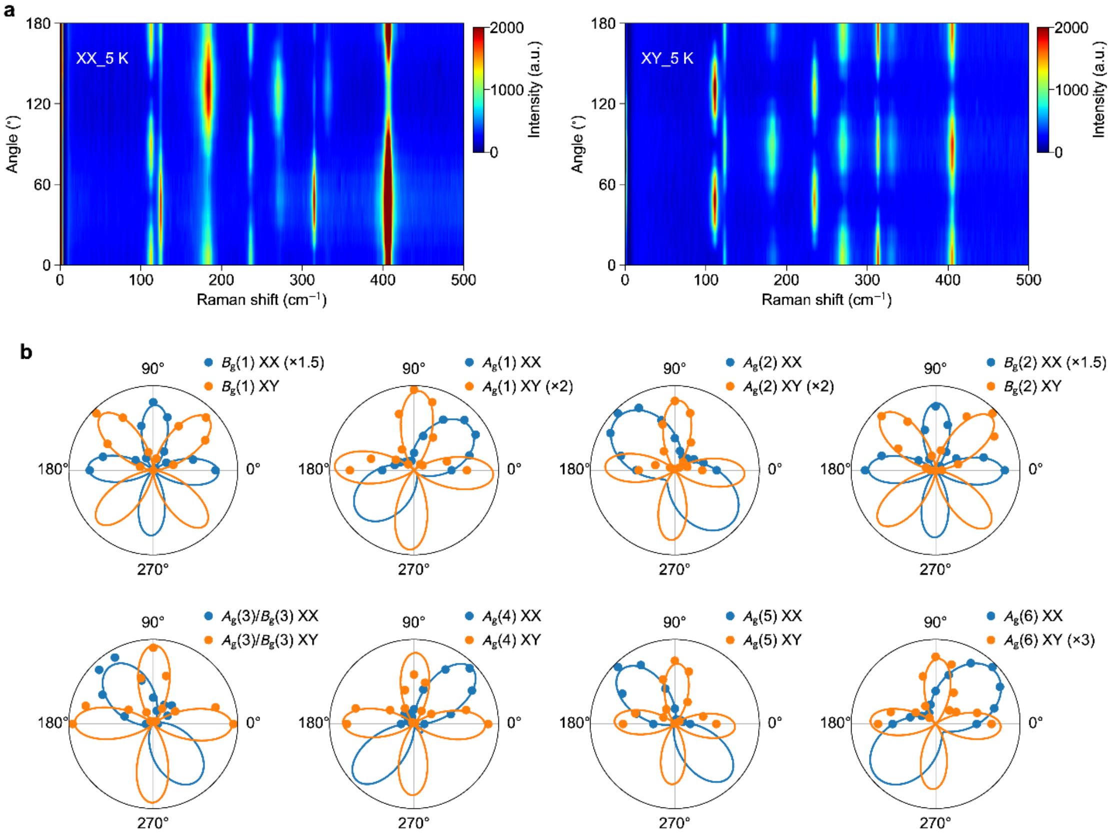

Supplementary Fig. 1| Phonon modes measured by polarization-angle dependent Raman spectroscopy. **a**, Polarization-angle dependent Raman scattering intensity maps at 5 K in the parallel (XX) and perpendicular (XY) polarization configurations. **b**, Polar plots of the angular dependence of the integrated intensity extracted from the Lorentzian-peak fitting, in the XX and XY polarization configurations for each phonon mode. The dots are experimental data and the solid lines are corresponding fitting results using the Eq. (6) in the main text. Please note that the deviation of the angle-dependence of the $A_g(3)$ mode in the XX polarization configuration is possibly due to relatively small contribution from the $B_g(3)$ mode, which is almost degenerate ($\Delta\omega \sim 0.3$ meV) with the $A_g(3)$ mode.

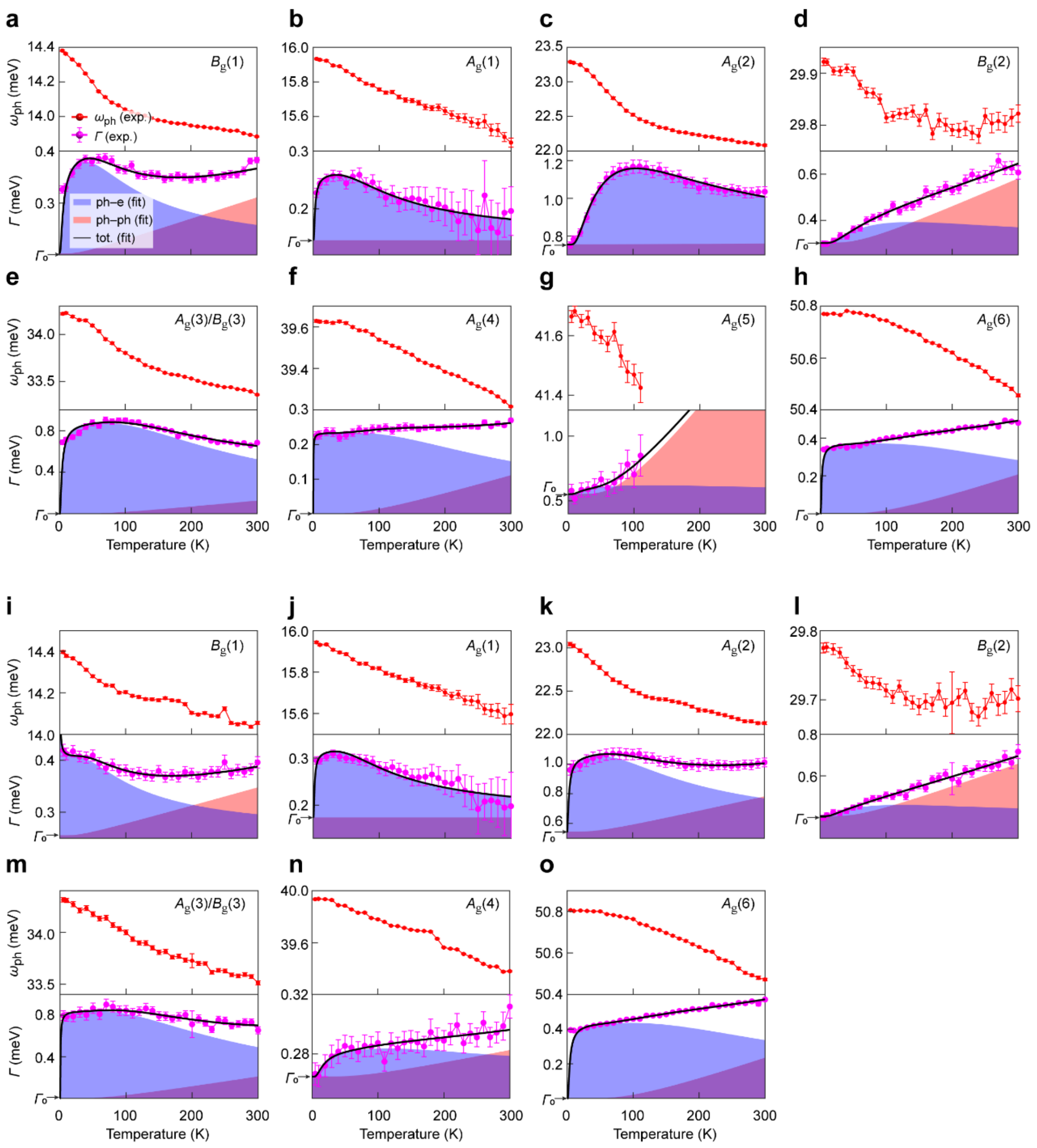

Supplementary Fig. 2| Fitting of the temperature-dependent Raman spectroscopy results. **a-h**, Upper panel: Fitted temperature-dependent phonon energies of 8 observed Raman-active phonon modes on sample S1. Lower panel: Fitted temperature-dependent phonon linewidths (half-width-at-half-maximum of the Lorentzian profile) of 8 observed Raman-active phonon modes on sample S1. The Raman spectra are fitted to Lorentzian functions as $I(\omega) = \frac{I_0}{1+\left(\frac{\omega-\omega_{\mathrm{ph}}}{\Gamma}\right)^2}$, where ω_{ph} is the phonon energy and Γ is the phonon linewidth. The temperature dependence of the linewidths is interpreted by combined contributions from phonon–electron scattering (blue shaded area) and phonon–phonon (red shaded area) based on Eq. (1) in the main text. The fitted results from these two scattering mechanisms are upshifted by a constant background Γ_0 (as indicated by black arrows) for a better illustration. **i-o**, Same as **a-h**, but measured on a different sample S2. Please note $A_g(5)$ is too weak to be resolved in sample S2. Error bars are standard deviations obtained from the Lorentzian fits to the phonon peaks.

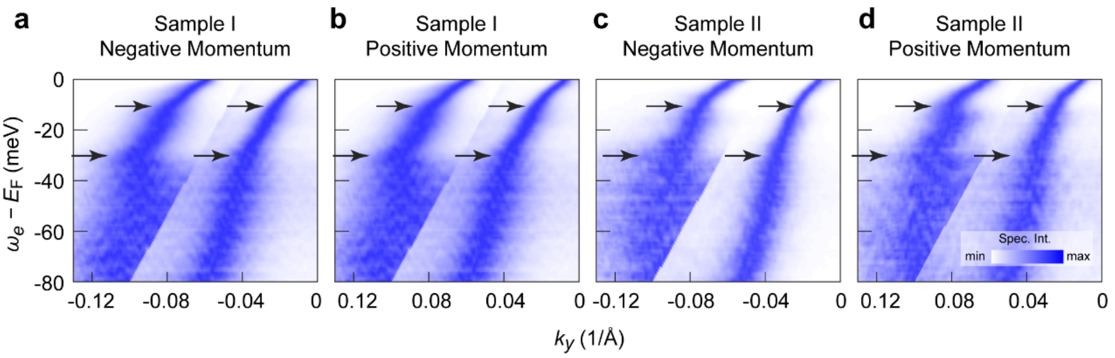

Supplementary Fig. 3| Reproducible kink features by ARPES. The observation of dispersion kinks in different samples (**a** and **c**, **b** and **d**) and in both positive and negative momentum branches (**a** and **b**, **c** and **d**).

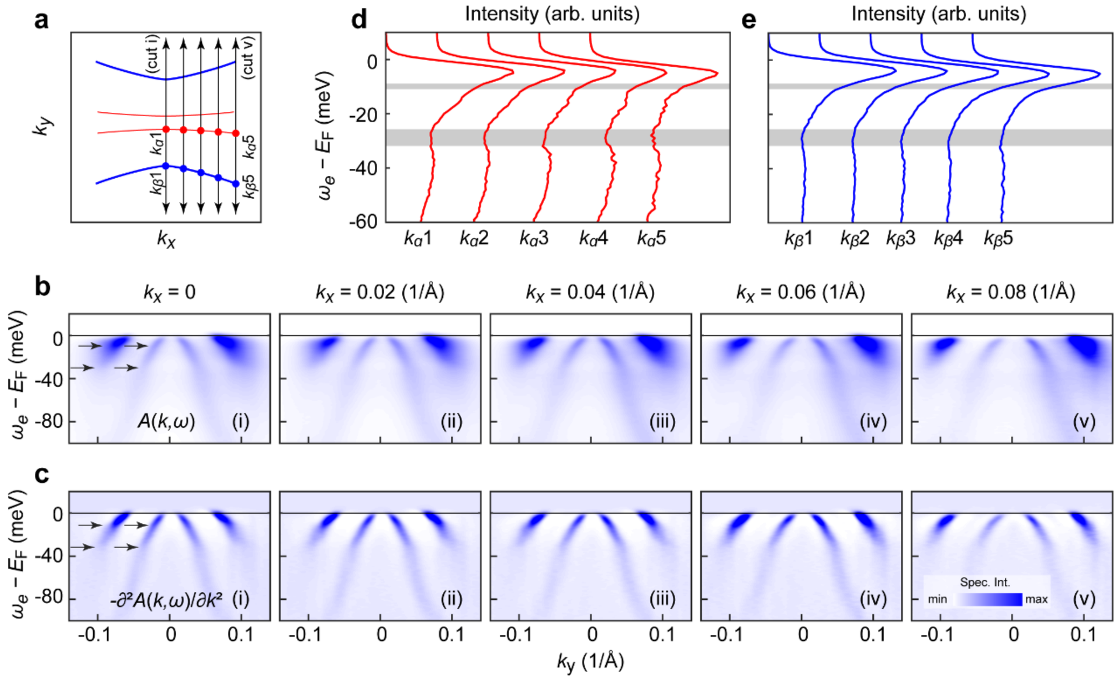

Supplementary Fig. 4 Momentum-dependent band dispersions by ARPES. **a**, Schematics of 2M-WS_2 Fermi surface. The topological surface state (TSS) and the bulk state (BS) are indicated by red and blue lines, respectively. **b**, ARPES measured band dispersion along momentum directions as indicated by the double-headed black arrows shown in **a**. **c**, Corresponding second-derivative intensity plots of **b** along the momentum-distribution-curve (MDC) direction. The kink features at ~10 and ~30 meV are marked by black arrows in **b** and **c**. **d**, Stacking plots of energy-distribution-curves (EDCs) at the Fermi momenta of the TSS, as indicated by the red dots in **a**. **e**, Stacking plots of energy-distribution-curves (EDCs) at the Fermi momenta of the BS, as indicated by the blue dots in **a**. The kink features at ~10 and ~30 meV are highlighted by the gray shaded areas in **d** and **e**.

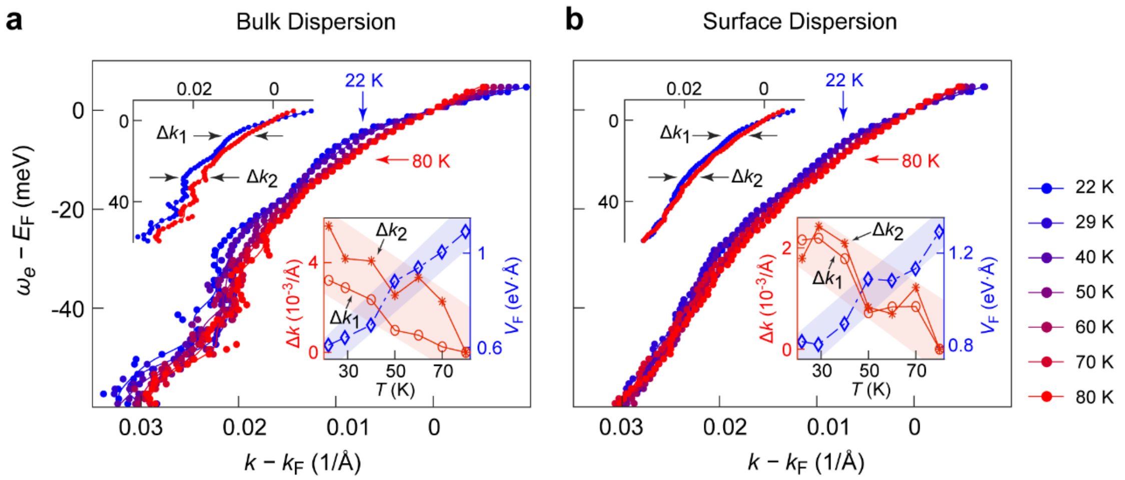

Supplementary Fig. 5| Temperature-dependent band dispersions by ARPES. **a**, Temperature-dependence of MDC-derived bulk band dispersion between 22 and 80 K. Top left inset: comparison of the dispersion measured at 22 K (blue curve) and 80 K (red curve). The momentum difference between these two dispersions at two kink energies are indicated as Δk_1 and Δk_2, respectively. Bottom right inset: temperature evolution of momentum difference (red curves) defined in the top left inset and Fermi velocity v_F (blue curve). **b**, Same as **a** but for the surface dispersion.

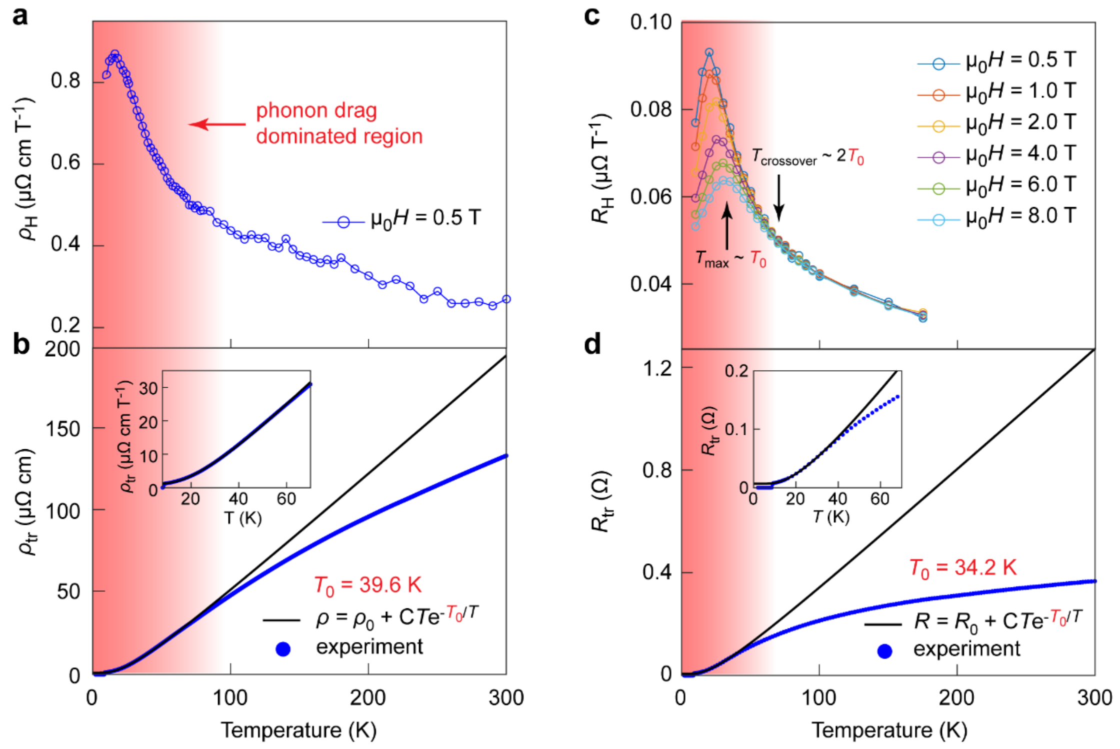

Supplementary Fig. 6| Phonon drag dominated transport properties. **a**, The temperature-dependent Hall resistivity from 10 to 300 K measured at $\mu_0 H$ = 0.5 T. **b**, The temperature-dependent resistivity from 2 to 300 K. The inset shows the zoom-in plot below 70 K which can be well fitted by a phonon–drag model $\rho = \rho_0 + CTe^{-T_0/T}$. The experimental data shown in **a** and **b** are extracted from Ref[1]. **c**,**d**, Same as **a**,**b** but measured on one of our samples. The magnetic field-dependent Hall resistivity peaks at $T_{\max} \sim T_0$ and the Hall resistivity show negligible magnetic field dependence above $T_{\mathrm{crossover}} \sim 2T_0$.

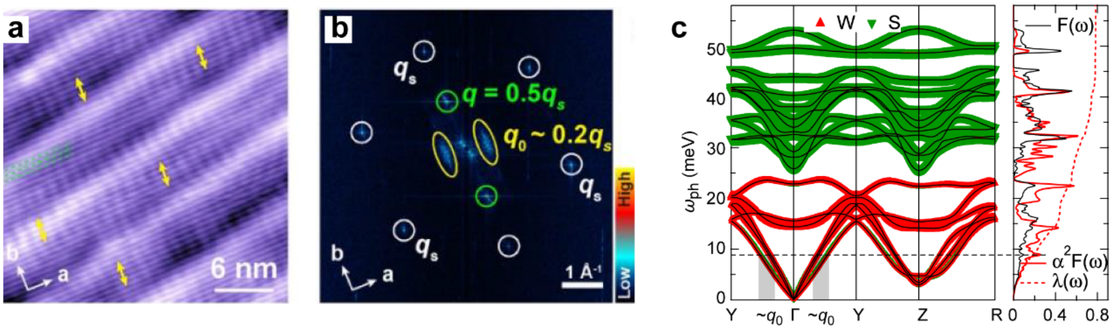

Supplementary Fig. 7| Possible origin of the stripe charge order due to EPC. **a**, Stripe modulation over a large area of 2M-WS_2 (30 nm × 30 nm; set point, $V_s = -5$ mV, $I_t = 100$ pA). The double-headed yellow arrows denote some valleys of the stripes. **b**, Fast Fourier transform result of **a**. The two parallel, line-features (near q_0) correspond to the stripe modulation in real space. The wavevectors q and q_S correspond to the zigzag chains of S atoms and the S-lattice, respectively. **c**, Bulk phonon spectrum, phonon DOS $F(\omega)$, Eliashberg spectral function $\alpha^2F(\omega)$, and accumulated $\lambda(\omega)$. The black dashed line denotes the energy of the acoustic phonons that contribute to the lowest-energy peak of $\alpha^2F(\omega)$. The momenta of these acoustic phonons are approximately q_0 (as indicated by the gray shaded areas). **a** and **b** are adapted with permission from Ref[2]. **c** is adapted with permission from Ref[3]. Copyright © 2020 American Chemical Society.

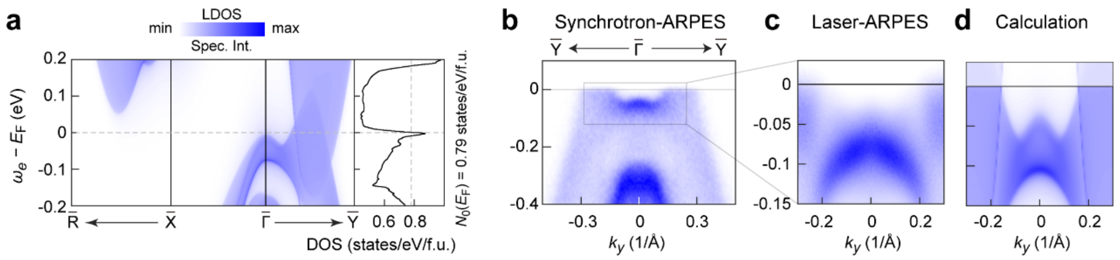

Supplementary Fig. 8| Band structure characterization of 2M-WSe₂. **a**, The calculated noninteracting band structure projected on the (100) surface (left panel) and corresponding DOS (right panel). **b**, Synchrotron-ARPES measured band dispersion along the $\overline{\mathrm{Y}}-\overline{\Gamma}-\overline{\mathrm{Y}}$ direction. **c**, Laser-ARPES measured band dispersion in the momentum-energy region as indicated by the gray rectangle in **b**. **d**. Corresponding band structure calculation in the same momentum-energy region as in **c**.

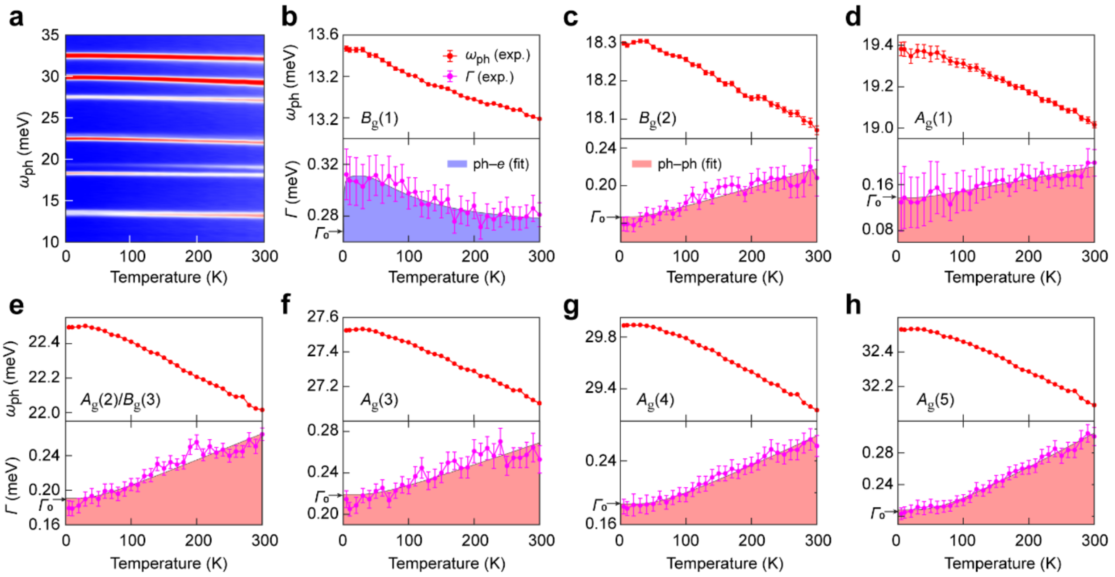

Supplementary Fig. 9| Raman spectroscopy characterization of 2M-WSe₂. a, Temperature-dependent Raman spectra from 5 to 300 K. **b-h**, Upper panel: Fitted temperature-dependent phonon energies of 7 observed modes based on Lorentzian functions. Lower panel: Fitted temperature-dependent phonon linewidths (half-width-at-half-maximum of the Lorentzian profile) of 7 observed modes. The fitted results are interpreted by either phonon–electron scattering (blue shaded area in **b**) or phonon–phonon scattering (red shaded area in **c-h**) based on Eq. (1) in the main text. The fitted results from these two scattering mechanisms are upshifted by a constant background Γ_0 (as indicated by black arrows) for a better illustration. Error bars are standard deviations obtained from the Lorentzian fits to the phonon peaks.

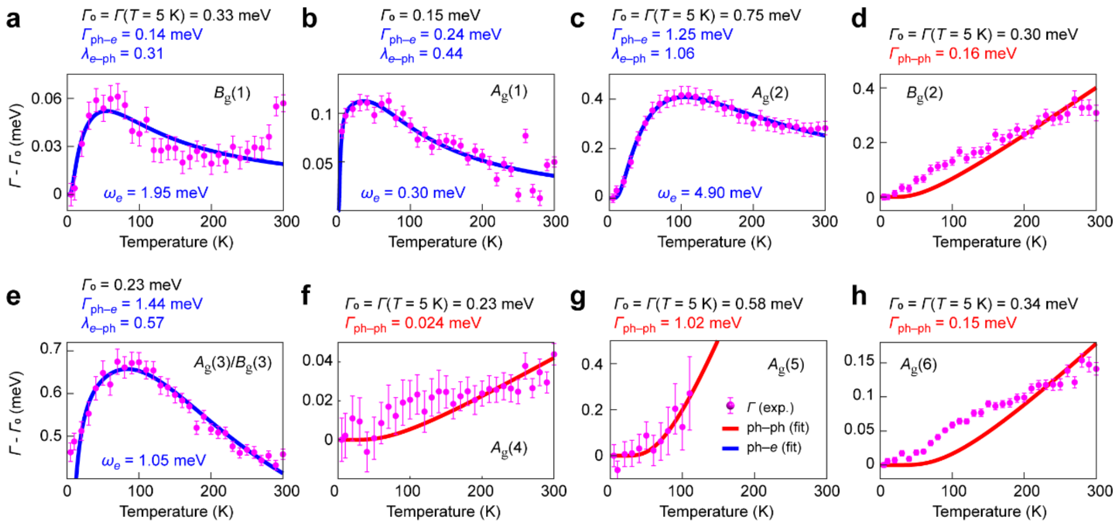

Supplementary Fig. 10| Fitted phonon linewidths of S1 based on phonon–electron or phonon–phonon scattering for the 8 Raman-active phonon modes of 2M-WS₂. a-c and e, The phonon linewidths of Raman modes $B_g(1)$, $A_g(1)$, $A_g(2)$ and $A_g(3)/B_g(3)$ are fitted based on phonon–electron scattering only. **d and f-h,** The phonon linewidths of Raman modes $B_g(2)$, $A_g(4)$, $A_g(5)$ and $A_g(6)$ are fitted based on phonon–phonon scattering only. Error bars are standard deviations obtained from the Lorentzian fits to the phonon peaks. Constraints of Γ_0 are applied. Please refer to Supplementary Note 1 for details.

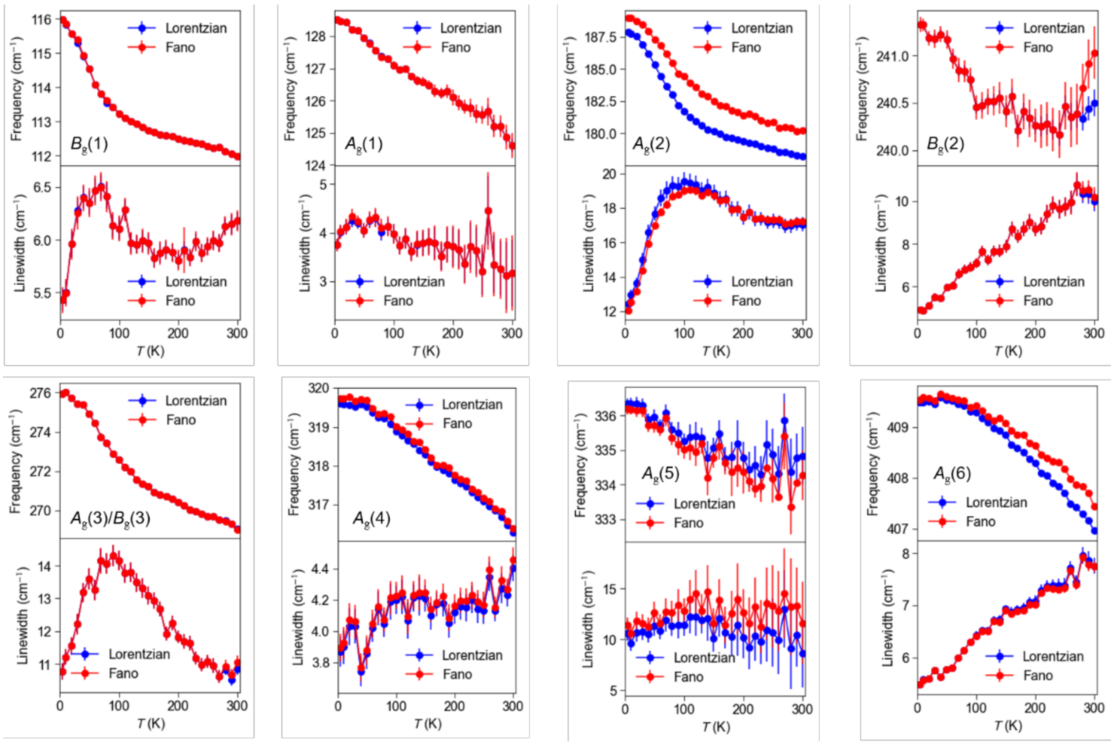

Supplementary Fig. 11| Comparison of fitted phonon frequencies and linewidths by using Lorentzian and Fano line shapes for all observed 8 Raman-active modes. Error bars are standard deviations obtained from the Lorentzian fits to the phonon peaks. Please refer to Supplementary Note 1 for details.

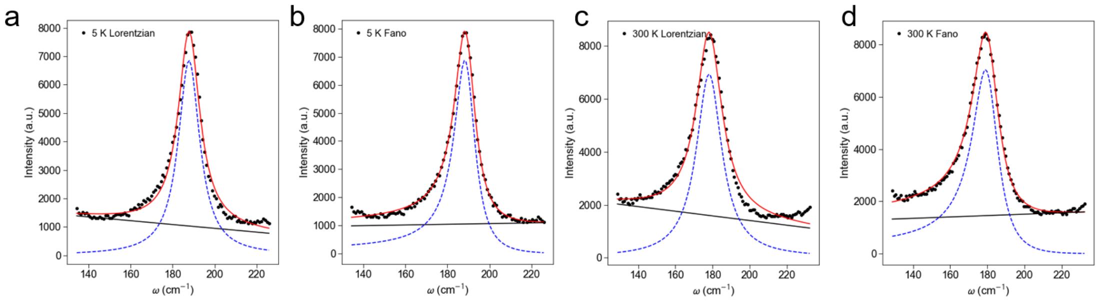

Supplementary Fig. 12| Lorentzian (a,c) and Fano (b,d) line profile fittings of Raman spectra of phonon mode $A_g(2)$ measured at 5 K (a,b) and 300 K (c,d).

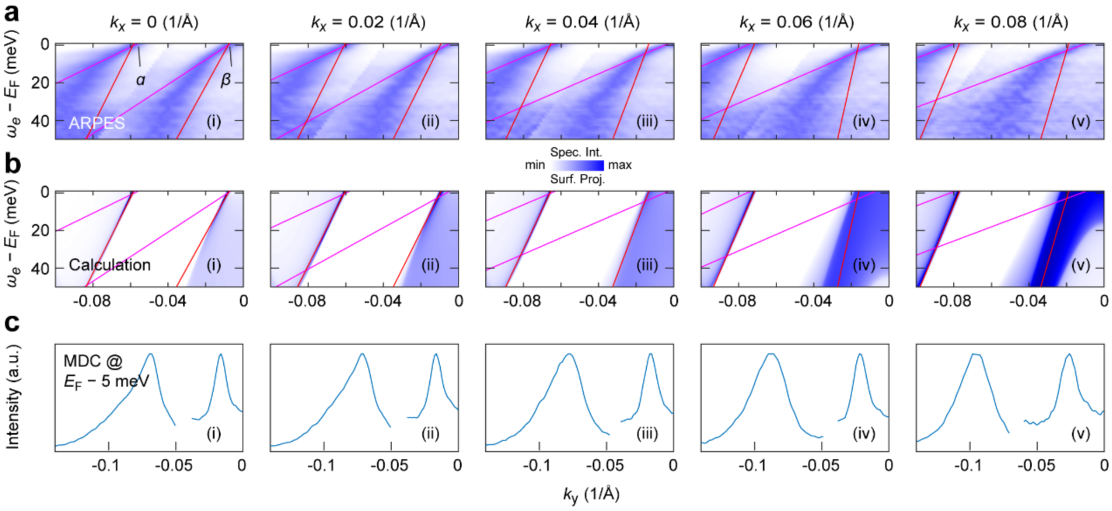

Supplementary Fig. 13| Fermi velocity renormalization along different momentum directions. a, ARPES measurements cutting with different k_x momenta. **b**, Corresponding non-interacting *ab initio* calculation. The magenta lines and red lines are linear fitting results of the local intensity maxima of **a** and **b** in the energy window between E_F and $E_F - 5$ meV. The slopes of these lines are defined as Fermi velocities. **c**, Momentum-distribution-curves (MDC) at $E_F - 5$ meV extracted from **a**. Please refer to Supplementary Note 2 for details.

k_x (1/Å)	v_F^α(exp.) (eV Å)	v_F^α(cal.) (eV Å)	$\frac{v_F^\alpha\text{(cal.)}}{v_F^\alpha\text{(exp.)}} - 1$	v_F^β(exp.) (eV Å)	v_F^β(cal.) (eV Å)	$\frac{v_F^\beta\text{(cal.)}}{v_F^\beta\text{(exp.)}} - 1$
0	0.49	2.00	3.1	0.65	1.81	1.9
0.02	0.46	2.00	3.3	0.55	2.00	2.6
0.04	0.43	2.12	4.0	0.43	2.64	5.1
0.06	0.46	2.32	4.0	0.44	4.49	9.3
0.08	0.38	2.42	5.4	0.38	3.41	8.1

Supplementary Table 1| Fitted Fermi velocities at different k_x momenta. $v_F^{\alpha(\beta)}$(exp.) and $v_F^{\alpha(\beta)}$(cal.) are fitted experimental and calculated Fermi velocities of $\alpha(\beta)$ band as indicated in Supplementary Fig. 13a. Please refer to Supplementary Note 2 for details.

Peak	$B_g(1)$	$A_g(1)$	$A_g(2)$	$B_g(2)$	$A_g(3)$/ $B_g(3)$	$A_g(4)$	$A_g(5)$	$A_g(6)$
$\omega_0^{\mathrm{cal.}}$ (meV)	14.4	16.0	22.5	29.3	33.5/33.8	38.4	39.9	50.7
$\omega_0^{\mathrm{Raman}}$ (meV) (S1)	14.4	15.9	23.3	29.9	34.2	39.6	41.7	50.8
$\omega_0^{\mathrm{Raman}}$ (meV) (S2)	14.4	15.9	23.0	29.8	34.3	39.9	N. A.	50.8
Γ_0^i (meV) (S1)	0.20	0.15	0.75	0.30	0.00	0.00	0.55	0.00
Γ_0^i (meV) (S2)	0.26	0.17	0.54	0.41	0.00	0.26	N. A.	0.00
$\Gamma_{\mathrm{ph-ph}}^i$ (meV) (S1)	0.017	0.00	0.001	0.109	0.060	0.064	0.796	0.174
$\Gamma_{\mathrm{ph-ph}}^i$ (meV) (S2)	0.015	0.00	0.007	0.098	0.099	0.010	N. A.	0.197
$\Gamma_{\mathrm{ph-}e}^i$ (meV) (S1)	0.41	0.24	1.23	0.28	1.82	0.47	0.16	0.76
$\Gamma_{\mathrm{ph-}e}^i$ (meV) (S2)	0.30	0.29	1.08	0.15	1.69	0.04	N. A.	0.90
ω_e^i (meV) (S1)	0.73	0.30	4.85	6.50	0.42	0.03	2.99	0.20
ω_e^i (meV) (S2)	-0.09	0.21	0.36	4.97	0.08	2.27	N. A.	0.41
$\lambda_{e\mathrm{-ph}}^i$ (S1)	0.912	0.438	1.045	0.145	0.718	0.138	0.042	0.136
$\lambda_{e\mathrm{-ph}}^i$ (S2)	0.663	0.530	0.939	0.077	0.663	0.011	N. A.	0.166

Supplementary Table 2| Complete fitting parameters extracted from the temperature-dependent Raman spectroscopy measurements. The temperature dependence of the Raman linewidths of each mode i is fitted based on Eq. (1) in the main text including 4 fitting parameters: Γ_0, $\Gamma_{\mathrm{ph-ph}}$, $\Gamma_{\mathrm{ph-e}}$, and ω_e. $\omega_0^{\mathrm{Raman}}$ is the phonon energies extracted from the lowest-temperature (5 K) Raman measurements by Lorentzian fitting. $\lambda_{e\mathrm{-ph}}^i$ is the mode-selective EPC strength derived from Eq. (2), where $N_0(E_F)$ = 1.38 states/eV/f.u. = 2.76 states/eV/u.c. is obtained from the noninteracting *ab initio* calculation (see Fig. 1g). f.u. represents formula unit and u.c. represents unit cell. The measurements were performed on 2 samples (S1, as shown in the main text, and S2). The detailed fitting results are presented in Supplementary Fig. 2.

Compounds	γ (mJ/mol/K^2)	β (mJ/mol/K^4)	$N^*(0)$ (states/eV/f.u.)	Θ_D (K)	T_C (K)	$\lambda_{e\text{-ph}}$
$Nb_{0.5}Ir_{0.5}Te_4$[4]	1.34	0.97	0.57	182	0	0
$MoTe_2$[5]	3.06	0.64	1.30	209	0.1	0.31
$PdTe_2$[6]	4.4	0.7	1.87	203	1.6	0.47
$Ni_{0.9}Re_{0.1}Te_2$[7]	5.02	0.97	2.13	182	2.3	0.53
$Ni_{0.8}Re_{0.2}Te_2$[7]	6.08	1.22	2.58	169	2.4	0.55
PdTeSe[8]	4.66	0.6	1.98	213	2.7	0.53
$Ir_{0.96}Pt_{0.04}Te_2$[9]	6.3	0.64	2.67	195	3	0.56
2M-WS_2[10]	8.97	0.49	3.81	228	8.8	0.80

Supplementary Table 3| Summary of the electronic specific heat coefficient (γ), the phonic specific heat coefficient (β), the renormalized DOS, the Debye temperatures (Θ_D), superconducting transition temperatures (T_C), and the EPC strengths ($\lambda_{e\text{-ph}}$) of the transition metal dichalcogenides (TMDs) presented in Fig. 4b in the main text. The low-temperature specific heat is contributed by electrons and phonons, which are proportional to T and T^3, respectively, as $C_v = \gamma T + \beta T^3$. The electronic specific heat coefficient (γ), also referred as the Sommerfeld coefficient is proportional to the renormalized DOS at E_F: $N(E_F) = \frac{3\gamma}{\pi^2 k_B^2}$, where k_B is the Boltzmann constant. The phonic specific heat coefficient (β) is associated with the Debye temperature: $\Theta_D = \sqrt[3]{\frac{12\pi^4 Rn}{5\beta}}$, where R is the ideal gas constant and n is the number of atoms per formula unit. The EPC strength ($\lambda_{e\text{-ph}}$) is derived by McMillan formula based on the superconducting transition temperature (T_C) and the Debye temperature (Θ_D) as $T_C = \frac{\Theta_D}{1.45}\exp\left[-\frac{1.04(1+\lambda_{e\text{-ph}})}{\lambda_{e\text{-ph}}-\mu^*(1+0.62\lambda_{e\text{-ph}})}\right]$, where $\mu^* = 0.1$ is the Coulomb parameter[4–10].

Supplementary Note 1. Inaccuracy of the EPC constant by Raman measurements

The inaccuracy of the EPC constant deduced by the fitting of Raman phonon linewidths is due to complicated origins, such as inaccurate fitting models, unstable fitting procedures, or improper choices of background. The inaccuracy of the fitting model has been discussed in the main text.

In our fitting procedure as shown in Supplementary Fig. 2, Γ_0 is one of the four free fitting parameters (Γ_0, $\Gamma_{\text{ph–ph}}$, $\Gamma_{\text{ph–e}}$, and ω_e). This results in satisfying fitting results of the temperature-dependent phonon linewidths. However, to improve the stability of the fitting procedure, less free fitting parameters are favorable.

For this purpose, we classify the 8 Raman-active phonon modes into two categories. The first category includes $B_g(1)$, $A_g(1)$, $A_g(2)$, and $A_g(3)/B_g(3)$ showing the nonmonotonic temperature-dependent behavior (see Supplementary Fig. 10a-c and e). The second category includes $B_g(2)$, $A_g(4)$, $A_g(5)$, and $A_g(6)$ showing the conventional monotonic increase of phonon linewidths with temperature (see Supplementary Fig. 10d and f-h). Phonon–electron scattering and phonon–phonon scattering dominate in these two categories, respectively. One can see that contributions from one mechanism of phonon–electron or phonon–phonon scattering can lead to decent fitting results for the 8 Raman-active phonon modes, as shown in Supplementary Fig. 10.

In this fitting procedure, constraints of Γ_0 are also applied. Based on Eq. (1) in the main text, the linewidth broadenings due to phonon–phonon and phonon–electron scattering converge to zero at zero temperature when $\omega_e > 0$. Therefore, we set $\Gamma_0 = \Gamma(T = 5\text{ K})$ for all modes except for $A_g(1)$ and $A_g(3)/B_g(1)$. For these two modes, the zero-temperature limit is inapplicable for $T = 5\ K$ since ω_e and k_BT are comparable. Γ_0 of all 8 Raman-active modes are of the same order of magnitude (0.23~0.75 meV) and zero Γ_0 are avoided. Both fitting procedure result in an estimation of the EPC constant of order 1.

As shown in Supplementary Fig. 11, we have checked the fitting results by using the Fano line shape for all 8 Raman-active phonon modes. One can see that Lorentzian and Fano line shape fittings result in perfectly consistent linewidths for all 8 modes. The fitted frequencies show small discrepancies (< 0.5 meV) only for $A_g(1)$ in the whole temperature range (5~300 K) and $A_g(6)$ in the high-temperature region ($T > 100$ K). The small differences in frequencies and linewidths between

Lorentzian and Fano fittings should have negligible influence (<1%) in the estimation of the total EPC constant, based on Eq. (2) in the main text.

The most pronounced asymmetric line profile has been observed in $A_g(2)$ mode, as shown in Supplementary Fig. 12. As asymmetric Fano line shape is an experimental indication for strong EPC, this observation is consistent with our fitting results showing in Supplementary Fig. 10c that $A_g(2)$ mode exhibits the strongest partial EPC constant (1.06) among all 8 Raman-active modes.

Supplementary Note 2. Momentum-dependent Fermi velocity renormalization

The renormalized Fermi velocities are quantitatively extracted along 5 different momentum directions, as indicated by the magenta lines in Supplementary Fig. 13a,b. Correspondingly, the "bare" Fermi velocities are extracted from the (non-interacting) *ab initio* calculation, as indicated by the red lines in Supplementary Fig. 13a,b. Surprisingly, this analysis approach leads to a clear variance of the coupling strength (1.9~9.3) for different bands and momentum directions, as shown in Supplementary Table 1.

However, we argue that the variance is not intrinsic and does not evidence momentum dependence of the many-body interaction for the following reasons. The above analysis approach can be inaccurate considering the mixture of the surface and bulk states. The band β is a topological surface state along k_x = 0, which is separated with the bulk continuum in the *k*-*E* space. Hence, it results in a reliable coupling strength (1.9). For momentum directions away from $\bar{\Gamma} - \bar{\mathrm{Y}}$ $(k_x \neq 0)$, the band β gradually merges into the bulk continuum. Similarly, the band α has increased bulk contribution for larger k_x. The bulk origins are experimentally evidenced by the momentum-distribution-curves (MDCs) with enhanced peak widths and asymmetric profiles, as shown in Supplementary Fig. 13c. The uncertainty of bulk dispersions due to k_z broadening can lead to unreliable results and significant overestimation of the coupling strength.

Based on the consideration above, we only performed the quantitative analysis for the band β along the $\bar{\Gamma} - \bar{\mathrm{Y}}$ direction (k_x = 0) in the main text. The momentum dependence of the many-body interaction is important for the understanding of the superconducting mechanism and can be relevant in 2M-WS_2, however, it requires further investigations.

Supplementary References

1. Yang, Y. *et al.* Anomalous enhancement of the Nernst effect at the crossover between a Fermi liquid and a strange metal. *Nat. Phys.* **19**, 379–385 (2023).
2. Fan, X. *et al.* Stripe charge order driven manipulation of Majorana bound states in 2M-WS_2 topological superconductor. Preprint at http://arxiv.org/abs/2308.16101 (2023).
3. Lian, C.-S., Si, C. & Duan, W. Anisotropic Full-Gap Superconductivity in 2M-WS_2 Topological Metal with Intrinsic Proximity Effect. *Nano Lett.* **21**, 709–715 (2021).
4. Zhou, W. *et al.* Nonsaturating Magnetoresistance and Nontrivial Band Topology of v Type ‐ II Weyl Semimetal $NbIrTe_4$. *Adv. Electron. Mater.* **5**, 1900250 (2019).
5. Chen, F. C. *et al.* Superconductivity enhancement in the S-doped Weyl semimetal candidate $MoTe_2$. *Appl. Phys. Lett.* **108**, (2016).
6. Salis, M. V., Huang, Y. K. & De Visser, A. Heat capacity of type-I superconductivity in the Dirac semimetal $PdTe_2$. *Phys. Rev. B* **103**, 104502 (2021).
7. Mandal, M. & Singh, R. P. Emergent superconductivity by Re doping in type-II Weyl semimetal $NiTe_2$. *J. Physics: Condens. Matter* **33**, 135602 (2021).
8. Liu, W. *et al.* Enhanced superconductivity in the Se-substituted 1T-$PdTe_2$. *Phys. Rev. Mater.* **5**, 014802 (2021).
9. Pyon, S., Kudo, K. & Nohara, M. Superconductivity Induced by Bond Breaking in the Triangular Lattice of $IrTe_2$. *J. Phys. Soc. Jpn.* **81**, 053701 (2012).
10. Wang, L. S. *et al.* Nodeless superconducting gap in the topological superconductor candidate 2M–WS_2. *Phys. Rev. B* **102**, 024523 (2020).
